# Immune Profiling of Gliomas Reveals a Connection with IDH1/2 Mutations, Tau Function and the Vascular Phenotype

**DOI:** 10.3390/cancers12113230

**Published:** 2020-11-02

**Authors:** Teresa Cejalvo, Ricardo Gargini, Berta Segura-Collar, Pablo Mata-Martínez, Beatriz Herranz, Diana Cantero, Yolanda Ruano, Daniel García-Pérez, Ángel Pérez-Núñez, Ana Ramos, Aurelio Hernández-Laín, María Cruz Martín-Soberón, Pilar Sánchez-Gómez, Juan M. Sepúlveda-Sánchez

**Affiliations:** 1 Instituto de investigación I+12, Hospital 12 de Octubre, 28041 Madrid, Spain; tcejagoy@uax.es (T.C.); diana_cantero@h12o.es (D.C.); yolanda_ruano@h12o.es (Y.R.); aurelio.hlain@salud.madrid.org (A.H.-L.); mmsoberon@salud.madrid.org (M.C.M.-S.); 2 Neurooncology Unit, Instituto de Salud Carlos III-UFIEC, 28220 Madrid, Spain; rgargini@isciii.es (R.G.); berta.segura@isciii.es (B.S.-C.); p.mata@isciii.es (P.M.-M.); beatriz.herranz@ufv.es (B.H.); 3 Facultad de Medicina de la Universidad Francisco de Vitoria, 28223 Madrid, Spain; 4 Dto. Neurocirugía, Hospital 12 de Octubre, Universidad Complutense, 28041 Madrid, Spain; dgarciaperez@salud.madrid.org (D.G.-P.); apnunez@salud.madrid.org (Á.P.-N.); 5 Dto. Radiología, Hospital 12 de Octubre, Universidad Complutense, 28041 Madrid, Spain; aramosgonzalez@salud.madrid.org

**Keywords:** gliomas, IDH mutations, immune profiling, tumor microenvironment, Tau

## Abstract

**Simple Summary:**

In the present work we have confirmed that gliomas with *isocitrate dehydrogenase 1/2* mutations are “cold” tumors, whereas the immune content of their wild-type counterparts is more heterogeneous. A large subgroup of wild-type glioblastomas is characterized by an important immune component, particularly enriched in myeloid cells, and an elevated expression of the ligand of programmed death ligand 1 (PD-L1) in the immune compartment. The rest contain few lymphocytes and myeloid cells. Notably, we have observed a direct correlation between the immune content and the presence of vascular alterations, as well as with the reduced expression of Tau, a microtubule-binding protein that we described as a negative regulator of angiogenesis. Using syngeneic mouse models, we show that overexpression of Tau reduces the immune content, delaying tumor growth.

**Abstract:**

Background: Gliomas remain refractory to all attempted treatments, including those using immune checkpoint inhibitors. The characterization of the tumor (immune) microenvironment has been recognized as an important challenge to explain this lack of response and to improve the therapy of glial tumors. Methods: We designed a prospective analysis of the immune cells of gliomas by flow cytometry. Tumors with or without *isocitrate dehydrogenase 1/2* (*IDH1/2*) mutations were included in the study. The genetic profile and the presence of different molecular and cellular features of the gliomas were analyzed in parallel. The findings were validated in syngeneic mouse models. Results: We observed that few immune cells infiltrate mutant *IDH1/2* gliomas whereas the immune content of *IDH1/2* wild-type tumors was more heterogeneous. Some of them contained an important immune infiltrate, particularly enriched in myeloid cells with immunosuppressive features, but others were more similar to mutant *IDH1/2* gliomas, with few immune cells and a less immunosuppressive profile. Notably, we observed a direct correlation between the percentage of leukocytes and the presence of vascular alterations, which were associated with a reduced expression of Tau, a microtubule-binding protein that controls the formation of tumor vessels in gliomas. Furthermore, overexpression of Tau was able to reduce the immune content in orthotopic allografts of GL261 cells, delaying tumor growth. Conclusions: We have confirmed the reduced infiltration of immune cells in *IDH1/2* mutant gliomas. By contrast, in *IDH1/2* wild-type gliomas, we have found a direct correlation between the presence of vascular alterations and the entrance of leukocytes into the tumors. Interestingly, high levels of Tau inversely correlated with the vascular and the immune content of gliomas. Altogether, our results could be exploited for the design of more successful clinical trials with immunomodulatory molecules.

## 1. Background

Gliomas are classified as lower-grade gliomas (LGGs) (grade II and III) or glioblastomas (GBMs) (grade IV). Among the first, detection of 1p/19q co-deletions discriminates oligodendrogliomas from astrocytomas, the latter being enriched in *α-thalassemia/mental retardation syndrome X-linked* (*ATRX*) mutations. By contrast, all GBMs have an astrocytic lineage and they accumulate diverse genetic alterations. Gliomas must be now classified based on the presence or absence of *isocitrate dehydrogenase 1/2* (*IDH1/2*) mutations, as this is a strong prognosis indicator [1].

In the last decade, immunotherapy with checkpoint inhibitors (ICIs) has been remarkably successful across several tumor types. By contrast, recent clinical trials using anti-programmed cell death 1 (PD-1) antibodies in recurrent GBM has shown very few responses [2], even though the ICIs seem to reach the brain [3]. Compared to responsive cancers, gliomas harbor a lower burden of somatic mutations, fewer infiltrative T cells and a more immunosuppressive tumor microenvironment (TME) [4,5,6]. These factors could explain why glial tumors remain largely refractory to ICIs. Nonetheless, gliomas are far from being a homogenous entity and disparities in their immune content might also condition their response to different therapeutic strategies. In order to classify the immunological profile of glial tumors, several groups have used computational and immunohistochemical (IHC) analysis approaches [7,8,9,10]. Here, we have performed a comprehensive characterization of the tumors by flow cytometry. Our results suggest that there is a correlation between the vascular phenotype and the entrance and/or the function of the hematopoietic cells on gliomas. Our group has recently described that Tau, a microtubule-binding protein, impairs the neovascularization of gliomas [11]. Here, we have shown that overexpression of Tau reduces the immune content in human samples and in orthotopic glioma models, modifying tumor growth. This knowledge suggests that vascular features could be used as a surrogate marker of the immune infiltrate and opens new venues to find synergistic therapeutic interventions.

## 2. Methods

### 2.1. Ethics Approval, Consent to Participate, and Data Availabilty

All patients (Table S1) gave written informed consent and the hole study was performed with the approval of the Ethical Committee at “Hospital 12 de Octubre” (CEI_14/023 and CEI_18/024). Consent for publication was not applicable since there are no personal data in the manuscript. All data generated or analyzed during this study are included in this published article [and its supplementary information files].

### 2.2. Molecular Characterization of the Tumors

The presence of IDH1 R132H mutations was tested by IHC staining on FFPE tissue sections with anti-IDH1 R132H (DIA H09; dilution 1:200; Dianova, Hamburg, Germany). Moreover, we used a custom Ampliseq (PCR-based) gene-targeted NGS (next generation sequencing) panel that analyzed 30 genes that were previously demonstrated to be frequently mutated in gliomas (including IDH1 and IDH2) [12]. DNA from formalin-fixed paraffin-embedded (FFPE) tumor tissues was extracted using the QIAamp DNA FFPE Tissue Kit (Qiagen, Venlo, Netherlands). DNA was quantified using a Qubit2.0 Fluorometer (Thermo Fisher Scientific, Waltham, MA, USA). Libraries were constructed from 10 ng of DNA using the Ion-AmpliSeq Library-Kit v2.0 (Thermo Fisher), according to the manufacturer′s instructions. Libraries were multiplexed, submitted to emulsion PCR and loaded into the chip using the Ion Chef System. Libraries were sequenced using Ion GeneStudio S5 system (Thermo Fisher Scientific) according to the manufacturer′s instructions, at average target panel coverage of 800X. O6-methylguanine-DNA-methyltransferase (MGMT) promoter methylation analysis was performed as previously reported [12].

### 2.3. Flow Cytometry Analysis

Tumor suspensions were obtained after mechanical and enzymatic disaggregation (Accumax (Merck Millipore, Burlington, MA, USA) (15 min, room temperature (RT)) and filtered through 70 μM nylon mesh cell strainer (Thermo Fisher Scientific). Erythrocytes were lysed with Quicklysis buffer (Cytognos, Salamanca, Spain) and cells were incubated with hFcR Blocking (Miltenyi Biotec, Bergisch Gladbach, Germany), previous to antibody (Table S2) incubation (20 min at 4 °C in PBS 1% fetal bovine serum (FBS)). Cells were labelled with a Fixable Viability Stain (Becton Dickinson, Franklin Lakes, NJ, USA) (20 min, RT). The analysis was conducted in a Macsquant10 flow Cytometry (Miltenyi Biotec). Subset definition was: Neutrophils: CD45^+^CD11b^+^CD16^+^CD15^+^CD14^−^CD33^−^; myeloid-derived suppressor cells (MDSCs): CD45^+^CD11b^+^CD16^+^CD15^−^CD14^+/−^CD33^+^; Macrophages: CD45^+^CD11b^+^CD16^−^CD15^−^CD14^+/−^CD33^−^MHCII^+^; Tregs: CD45^+^CD3^+^CD4^+^CD25^+^CD127^lo^.

### 2.4. Western Blot

The protein extract was generated by mechanical disaggregation using lysis buffer (50 mM Tris-pH 7.5, 300 mM NaCl, 0.5% SDS, and 1% Triton X-100) (15 min with agitation at 95 °C). Protein content was quantified using BCA Protein Assay Kit (Thermo Fisher Scientific,) and 20 µg of protein was resolved by 10% or 12% SDS-PAGE and then transferred to a nitrocellulose membrane (Amersham Biosciences, Little Chalfont, UK). The membranes were blocked (1 h, RT in PBS and 0.1% Tween-20 with 5% skimmed milk) and then incubated with the primary (Table S2) (overnight 4 °C) and the secondary (HRP-conjugated anti-mouse or -rabbit, Agilent Technologies, Santa Clara, CA, USA) (2 h, RT) antibodies diluted in PBS-T. Proteins were visualized with ECL (Bio-Rad Laboratories, Hercules, CA, USA) using the Imager 680 (Amersham).

### 2.5. Immunohistochemistry (IHC)

Samples were fixed in 10% formalin overnight, dehydrated through a series of graded ethanol baths and then infiltrated with paraffin. Then, 5-µm-thick sections were obtained in a microtome and then sections were rehydrated and permeabilized (1% triton X-100). Antigen retrieval was performed with Citrate Buffer (10 mM, pH 6) in a pressure cooker (2 min). Endogenous peroxidase inhibition and blocking with normal horse serum was also performed before the incubation with primary antibodies (Table S2) (overnight, 4 °C) and biotinylated secondary antibodies (15 min). Sections were then incubated with SAV-HRP (10 min) and with DAB (3 min). The IHC score was judged from 0 (no staining) to 4 (the strongest positive staining) in 10 high magnification pictures from each sample. For the quantification of the vasculature, we counted the number of dilated vessels per high-magnification field and the relative area covered by the CD34 positive staining. For the latter, 6 fields per sample were counted using the ImageJ program and applying the vascular density plugin.

### 2.6. qRT-PCR Assay

RNA was extracted from the tissue using an RNA Isolation Kit (Roche, Basel, Switzerland). Total RNA (1 µg) was reverse transcribed with a PrimeScript RT Reagent Kit (Takara Bio Inc., Kusatsu, Japan). Quantitative real-time PCR was performed using the Light Cycler 1.5 (Roche) with the SYBR Premix Ex Taq (Takara) and specific primers for each gene (Table S3). Gene expression was quantified by the delta-delta Ct method.

### 2.7. Cell Culture

The GL261 murine glioma cells were maintained in DMEM plus 10% FBS, 2mM L-glutamine, 0.1% penicillin (100 U/mL) and streptomycin (100 μg/mL). GL261 murine glioblastoma cells were obtained from the NCI-Frederick Cancer Research Tumor Repository (Frederick, MD, USA).

### 2.8. Lentivirus Preparation and Infection

Pseudotyped lentivectors were produced using reagents and protocols as previously described [1] and Addgene lentiviral protocols [11]. GL261 cells were infected for 48 h adding the lentiviral supernatant (LV-GFP and LV-Tau) and 4 μg/mL of polybrene. These GL261 cells were implanted into C57/BL6 mice.

### 2.9. Mouse Model Study

Animal experiments were reviewed and approved by the Research Ethics and Animal Welfare Committee at “Instituto de Salud Carlos III” (PROEX 244/14 and 02/16), in agreement with the European Union and national directives. Intracranial transplantations to establish orthotopic allo-grafts were performed injecting 50,000 cells (resuspended in 2 μL of culture medium) with a Hamilton syringe into the striatum of C57Bl/6 mice (A–P, −0.5 mm; M–L, +2 mm, D–V, −3 mm; related to Bregma) using a Stoelting Stereotaxic device.

### 2.10. In-Silico Studies

For studies of gene expression and gene profiling, the cancer genome atlas (TCGA) merged dataset (LGG+GBM) was used with 1153 enrolled patients and a set of 702 patients with LGG and GBM tumors with RNAseq values (IlluminaHiSeq). The different immune cell signatures (Activated CD8 cell, Central memory CD4 cell, Central memory CD8 cell, Regulatory cell, Type 1 helper cell, Type 2 helper cell, Macrophages, MDSC and Neutrophils) were obtained from [13]. For the GSEA (Gene Set Enrichment Analysis) study, the Tau/MAPT expression was continuously computed through the LGG+GBM dataset using the expression by RNAseq, then we used the method, continuous class label, and gene sets from the “CGP: chemical and genetic perturbations” and “CP (Canonical Pathways): BioCarta gene sets”. For correlation studies, the expression values were obtained from xena-browser RNA-seq dataset (TCGA merge LGG+GBM *n* = 702), who were filtered on the sequenced data (*n* = 661). For gene ontology analysis, the DAVID gene ontology 6.8 program was used (*n* = 500 genes).

### 2.11. Statistical Analysis

The quantified data were represented as mean ± SEM, compared between two groups using the two-tailed Student’s *t*-test. Differences are presented with statistical significance or p-value (* *p* < 0.05; ** *p* < 0.01; *** *p* < 0.001 and ns, not significant). For the correlation analysis between each protein, we used Pearson’s correlation coefficient (R2). An ANOVA test was used to compare multiple groups and contingency graphs (Chi-square tests) were performed for the analysis of the vascular and cellular scores in the tumors. P-values were calculated using the GraphPad program. For survival analysis, we used the Kaplan–Meier method and log-rank test using the SPSS program.

### 2.12. Data Availability

All data generated or analyzed during this study are included in this published article (and its supplementary information files).

## 3. Results

### 3.1. Stratification of Gliomas Based on the Immune Profile

**Figure 1:**
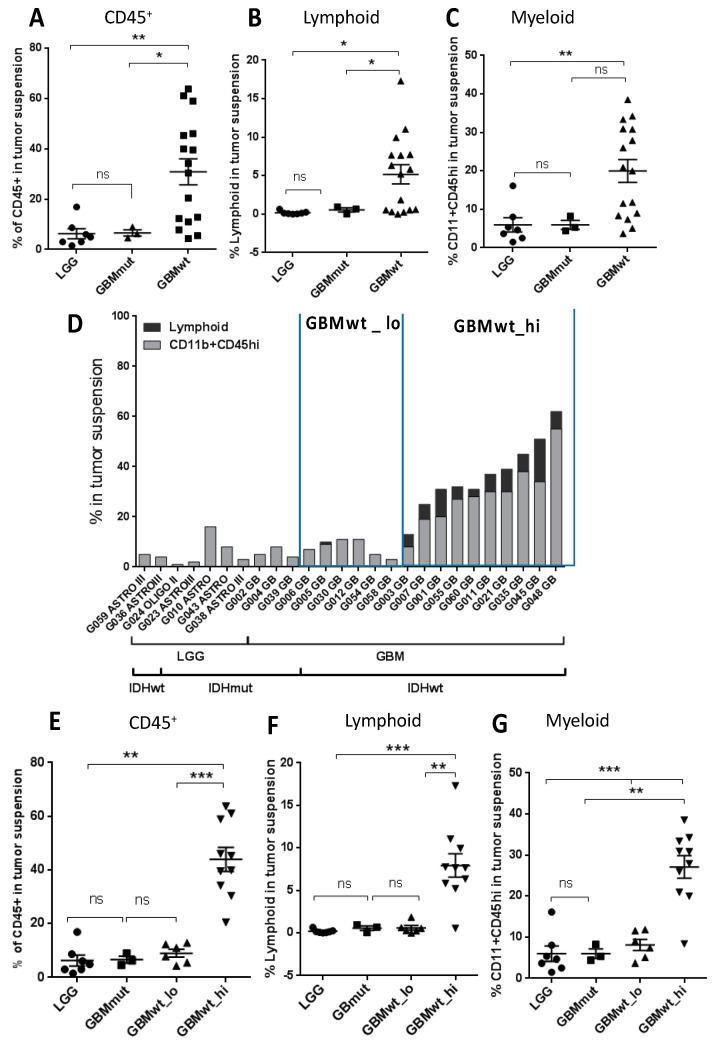
Flow cytometry analysis of the immune infiltrate of gliomas. (**A**–**C**) Percentage of CD45^+^ (**A**), lymphoid (CD45^+^CD11b^−^SSC^lo^) (**B**) and myeloid (CD45^+^CD11b^+^SSC^lo^ or SCC^hi^) (**C**) cells on total tumor suspensions in lower-grade glioma (LGG) (circles) (*n* = 7) and glioblastoma (GBM)-expressing mutant IDH (GBMmut) (triangles in A and squares in B and C) (*n* = 3) and wild-type IDH (GBMwt) (squares in A and triangles in B and C) (*n* = 16). (**D**) Percentage of lymphoid or CD11b^+^CD45^hi^ populations on total tumor suspension in individual samples. (**E**–**G**) Percentage of CD45^+^ cells (**E**), lymphoid (CD45^+^CD11b^−^SSC^lo^) (**F**) and myeloid (CD45^+^CD11b^+^SSC^lo^ or SCC^hi^) (**G**) cells on total tumor suspensions in the four groups of gliomas: LGG (circles) (*n* = 7), GBMmut (squares) (*n* = 3), GBMwt_lo (triangles) (*n* = 6), GBMwt_hi (inverted triangles) (*n* = 10). * *p* < 0.05, ** *p* < 0.01, *** *p* < 0.001, ns: not significant.

Samples from 28 patients diagnosed with glioma (Table 1) were dissociated and analyzed by flow cytometry (individualized data in Table S1). Tumors were classified based on the histology (GBM vs. LGG) and based on the presence of IDH mutations. As expected, the majority of LGGs were IDHmut (7/9), whereas only 3 out of 19 GBMs carried these mutations. The gating strategy described in Figure S1 was used to characterize different immune populations. In agreement with the literature [14], there was a significant increase in the number of CD45+ cells in IDHwt GBMs compared to IDHmut GBMs or to LGGs (Figure 1A). This increase was observed in both the lymphoid (Figure 1B) and the myeloid (Figure 1C) components. Among the IDHwt GBMs, we observed a heterogeneous profile: some of the tumors showed a low content of CD45+ cells, with a very similar percentage of immune cells to the one measured in IDHmut tumors (either LGGs or GBMs), whereas in others there was a strong presence of leukocytes, reaching 50% of the tumor content in some cases (Figure 1D). In order to find possible differences that could explain their distinct leukocyte extravasation, we decided to separate the IDHwt GBMs into two subgroups. Those GBMs that contained higher numbers of CD45+ cells (more than 10% of the cellular suspension) and increased amounts of lymphocytes (more than 1% of the cellular suspension) were included in the GBMwt_hi subgroup, and the rest of the tumors in the GBMwt_lo subgroup. As a confirmation, the average content of CD45+ (Figure 1E), lymphoid (Figure 1F) and myeloid (Figure 1G) cells was clearly enriched in the first group compared to the rest of gliomas.

We then performed an analysis of the genetic profile of the tumors. As expected, *epidermal growth factor receptor (EGFR)* alterations were enriched in IDH1wt GBMs, whereas *TP53* mutations were common among the IDH1mut gliomas (Figure S2A). However, we did not detect clear differences between the genetic profile of GBMwt_lo and GBMwt_hi tumors. Regarding the clinical data (Table 1), patients carrying *IDH* mutations were significantly younger (*p* = 0.003) (IDHmut: median age: 40 years, range: 30–76 years; GBMwt_lo: median age: 57 years, range: 42–70 years; GBMwt_high: median age: 65 years, range: 38–82 years) and survived longer than their wild-type counterparts (Figure S2B). However, there was no significant differences in the clinical behavior of patients from both GBMwt immune subgroups (Figure S2B).

### 3.2. Characterization of the Myeloid and the Lymphoid Compartments in the Different Subgroups of Gliomas

**Figure 2:**
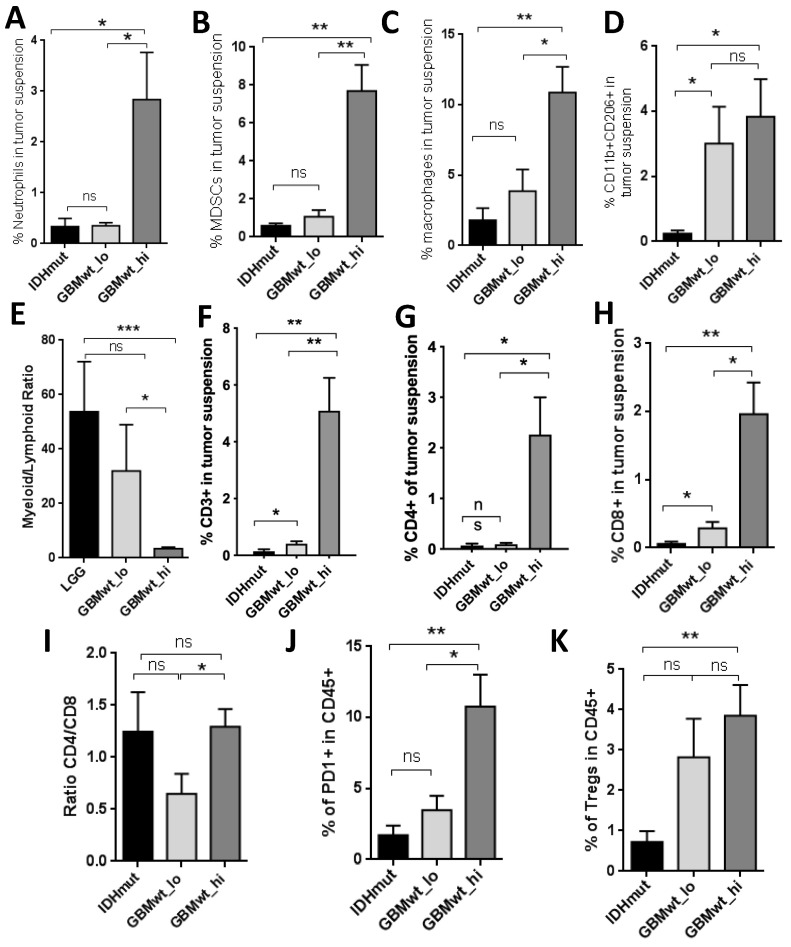
Proportions of myeloid and T cell subsets in glioma samples. (**A**–**C**) Percentage of neutrophils (**A**), myeloid-derived suppressor cells (MDSCs) (**B**) and macrophages (**C**) on total tumor suspensions from IDHmut (IDHmut LGG and GBM) (*n* = 6), GBMwt_lo (*n* = 5), and GBMwt_hi (*n* = 6) gliomas. (**D**–**E**) Percentage of CD206^+^ myeloid cells (**D**) and myeloid to lymphoid ratio (**E**) on total tumor suspensions from IDHmut (*n* = 4), GBMwt_lo (*n* = 6), and GBMwt_hi (*n* = 10) gliomas. (**F**–**I**) Percentage of T cells (**F**), CD4^+^ T cells (**G**) and CD8^+^ T cells (**H**) and ratio of CD4^+^ to CD8^+^ T cells (**I**) on total tumor suspensions from IDHmut (*n* = 4), GBMwt_lo (*n* = 6), and GBMwt_hi (*n* = 10) gliomas. (**J**–**K**) Percentage of PD1^+^ T cells (**J**) and T regs (**K**) within the CD45^+^ tumor subset of IDHmut (*n* = 5), GBMwt_lo (*n* = 6), and GBMwt_hi (*n* = 7) gliomas. * *p* < 0.05, ** *p* < 0.01, *** *p* < 0.001, ns: not significant.

In order to gain further insight into the composition of the immune infiltrate in the glioma subgroups, we dissected out the myeloid component in the tumor suspension. We combined the LGG and GBM IDH1mut (herein called IDHmut) for the subsequent comparisons with the other two groups of IDHwt GBMs. We observed that the percentage of neutrophils (Figure 2A), myeloid-derived suppressor cells (MDSCs) (Figure 2B) and macrophages (Figure 2C) was increased in GBMwt _hi compared to both IDHmut and GBMwt_lo gliomas. We also analyzed the presence of CD206, a typical marker of alternatively activated (M2) myeloid cells. We found a higher proportion of CD11b+CD206+ cells in GBMwt_lo and GBMwt_hi compared to IDHmut gliomas (Figure 2D). Our panel was not designed to recognize specifically resident microglia, but we found no differences in the transcription of *P2RY12*, which is highly expressed on microglia, between the three subgroups (Figure S3A). Moreover, ionized calcium binding adaptor molecule 1 (IBA1) positive cells were detected in high proportion in all the tumors analyzed (Figure S3B). The number of microglial cells (Figure S3C), as well as the total amount of IBA1 protein (Figure S3D–E), was very homogenous among the different gliomas. These results suggest that the main differences in the immune compartment of the distinct glioma subgroups are due to the entrance of cells from the blood. Notably, the ratio of myeloid to lymphoid cells was lower in GBMwt_hi compared to the other two subgroups (Figure 2E), suggesting that T cells infiltrate this subgroup of gliomas in particular. In agreement with that, we observed that the proportion of T cells (CD3+) (Figure 2F), in particular the CD4+ subset (Figure 2G) was higher in GBMwt_hi tumors than in the other two subclasses. However, there was an increase in the percentage of CD3+ cells in GBMwt_lo compared to IDHmut gliomas (Figure 2F). Furthermore, there was no difference between the percentages of CD8+ cells between the two subgroups of GBMs (Figure 2H), which was higher in both compared to IDHmut tumors. As a result, the CD4/CD8 ratio was lower in the GBMwt_lo compared to GBMwt_hi tumors (Figure 2I). This ratio has been linked to the appropriate lymphocyte function in other types of cancer [15]. Moreover, the proportion of PD1+ cells, which labels T cell exhaustion, was higher in GBMwt_hi compared to GBMwt_lo gliomas (Figure 2J), whereas the amount of regulatory T cells (Tregs) was similar in the two groups (Figure 2K). By contrast, IDHmut gliomas presented fewer exhausted T cells (Figure 2J) and Tregs (Figure 2K). Taken together, these findings highlight the important dissimilarities in the immune profile of IDHmut vs. IDHwt gliomas and suggest that the GBMwt_lo subgroup resembles IDHmut gliomas in their percentage of myeloid cells. Besides, we found differences in the lymphocyte content and function between the two subgroups of GBMs.

### 3.3. Enrichment of Programmed Death Ligand 1 (PD-L1) Expression in the Immune Cells of Highly Infiltrated Gliomas

**Figure 3:**
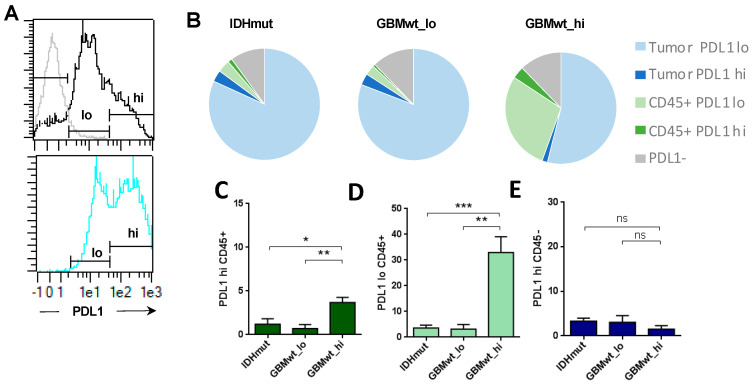
Programmed death ligand 1 (PD-L1) expression in gliomas. (**A**) The histograms show the isotype control labelling in grey line (negative) and low (lo) and high (hi) levels of expression of PD-L1 in the total tumor suspension (top, black line), or in the CD45+ subset (bottom, blue line) of a representative GBMwt_hi tumor. (**B**) The tart diagrams show the percentage of PD-L1^lo^ and PD-L1^hi^ tumor cells (CD45^−^) and leukocytes (CD45^+^) on total tumor suspensions in each glioma subgroup: IDHmut (*n* = 6), GBMwt_lo (*n* = 4) GBMwt_hi (*n* = 5). (**C**–**E**) Percentage of PD-L1^hi^CD45^+^ (**C**), PD-L1^lo^CD45^+^ (**D**) and PD-L1^hi^CD45^−^ (**E**) in each glioma subgroup. * *p* < 0.05, ** *p* < 0.01, *** *p* < 0.001, ns: not significant.

Our flow cytometry analysis in gliomas revealed two levels of expression of PD-L1 (herein defined as PD-L1_lo and PD-L1_hi) (Figure 3A), both in tumor (CD45−) and in immune (CD45+) cells. The expression profile was similar in GBMwt_lo and IDHmut tumors and very different from the GBMwt_hi gliomas (Figure 3B), which showed a strong increase in the percentage of CD45/PDL1 double positive cells. The increment was significant in both the PD-L1_hi (Figure 3C) and the PD-L1_lo (Figure 3D) populations. Notably, there were no differences in the amount of tumor cells expressing high levels of PD-L1 among the different subgroups of gliomas (Figure 3E). Altogether, our data suggest that there is a subgroup of IDHwt GBMs that contain a high immune infiltrate, enriched in myeloid cells and with a strong immunosuppressive profile: high content of MDSCs, CD206+ myeloid and PD-L1+ cells.

### 3.4. The Immune Stratification of the Tumors Correlates with Different Vascular Phenotypes

It has been proposed that the three different transcriptomic subtypes of gliomas (Proneural, PN, Classical, CL and Mesenchymal, MES) are associated with a different immune microenvironment [7]. When we analyzed our cohort of gliomas using qRT-PCR, we noticed that, as expected, PN and MES transcripts were enriched (Figure S4A–C) and diminished (Figure S4D–F), respectively, in IDHmut gliomas compared to their wild-type counterparts. However, there were no differences in the expression of PN (Figure S4A–C) or MES (Figure S4D–F) markers between the two subgroups of IDHwt GBMs. Therefore, neither the genetic (Figure S2A) nor the expression profiles seem to explain the existence of two distinct immune patterns in the aggressive IDHwt gliomas.

**Figure 4:**
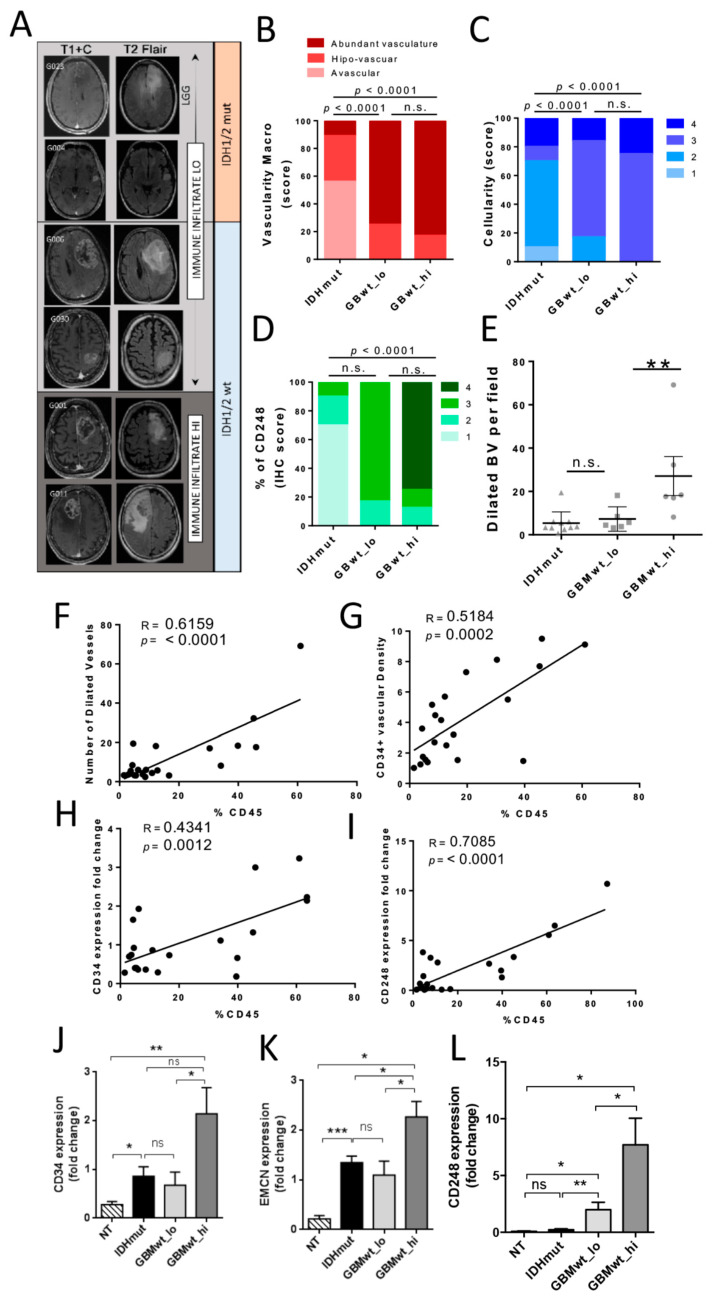
Correlation between the immune infiltrate and the vascular alterations in gliomas. (**A**) Representative magnetic resonance imagings (MRIs) (T1 post contrast (T1+C) and T2 FLAIR) of gliomas with low (LO) or high (HI) immune infiltrate. (**B**) Quantification of the blood vasculature score (macroscopic evaluation of the tumor vascularization (blood vessel (BV) macro) during surgery) in IDHmut (*n* = 9), GBMwt_lo (*n* = 6) GBMwt_hi (*n* = 8) tumors. (**C**–**E**) Quantification of the cellularity (estimated in the Hematoxilin & Eosine stainings) (**C**), the CD248 score (**D**), and the number of dilated blood vessels (BV) (**E**) in IDHmut (triangles) (*n* = 9), GBMwt_lo (squares) (*n* = 6) GBMwt_hi (circles) (*n* = 8) tumors. (**F**–**G**) Correlation between the percentage of CD45^+^ cells and the number of dilated BVs (**F**) and the density of CD34 staining (**G**) in the tissue sections (circles represent each individual tumor) (*n* = 21). (**H**–**I**) Correlation between the percentage of CD45^+^ cells and levels of *CD34* (**H**) and *CD248* (**I**) transcription measured by qRT-PCR analysis (circles represent each individual tumor) (*n* = 21). (**J**–**L**) qRT-PCR analysis of *CD34* (**G**), *EMCN* (H), and *CD248* (**I**) expression in normal tissue (NT) (*n* = 6) and in the three different glioma subgroups: IDHmut (*n* = 9), GBMwt_lo (*n* = 6) and GBMwt_hi (*n* = 8). *HPRT* transcription was used for normalization. * *p* < 0.05, ** *p* < 0.01, *** *p* < 0.001, ns: not significant.

In order to find disparities between the two subgroups of IDHwt GBMs that could correlate with their distinct immune landscapes, we performed a macroscopic analysis of the tumors. Preoperative magnetic resonance imaging (MRI) revealed clear differences between IDHmut and IDHwt gliomas, especially in the contrasting enhanced sequences (Figure 4A) [16]. However, the T1+C and T2 images of GBMwt_lo tumors were very similar to the ones obtained in the immune-high GBM subgroup (Figure 4A). In agreement with that, the macroscopic analysis of different vascular features revealed that the blood vasculature score (unbiased annotations from neurosurgeons) (Figure 4B) and the cellularity (Figure 4C) of the tumors were not significantly different in the two subgroups of GBMwt tumors. By contrast, we observed a slight increase in the CD248 IHC staining (Figure 4D and Figure S5), which labels tumor-pericytes in gliomas [17]. Accordingly, the number of vessels with a large lumen (herein called dilated blood vessels (BVs)) were higher in the GBMwt_hi tumors (Figure 4E and Figure S5). Moreover, the number of dilated vessels (Figure 4F), as well as the CD34 density (Figure 4G), correlated with the percentage of CD45+ cells measured by flow cytometry. To obtain an independent confirmation of these results, we performed a qRT-PCR analysis. We found a strong correlation between the CD45 content and the expression of the endothelial marker *CD34* (Figure 4H), as well as with the expression of *CD248* (Figure 4I). Moreover, the transcription of *CD34* (Figure 4J), *EMCN* (another marker of endothelial tumor cells) (Figure 4K) and *CD248* (Figure 4L) was increased in the GBMwt_hi group compared to the rest of gliomas. Notably, only the expression of *CD248*, was increased in GBMwt_lo tumors compared to IDHmut gliomas (Figure 4L), which correlated with the higher CD248 score measured by IHC (Figure 4D and Figure S5), suggesting a direct correlation between the absence of IDH mutations and the increase in tumor pericytes, as we have recently described [11].

### 3.5. Inverse Correlation of the Immune Content with Tau Expression

**Figure 5:**
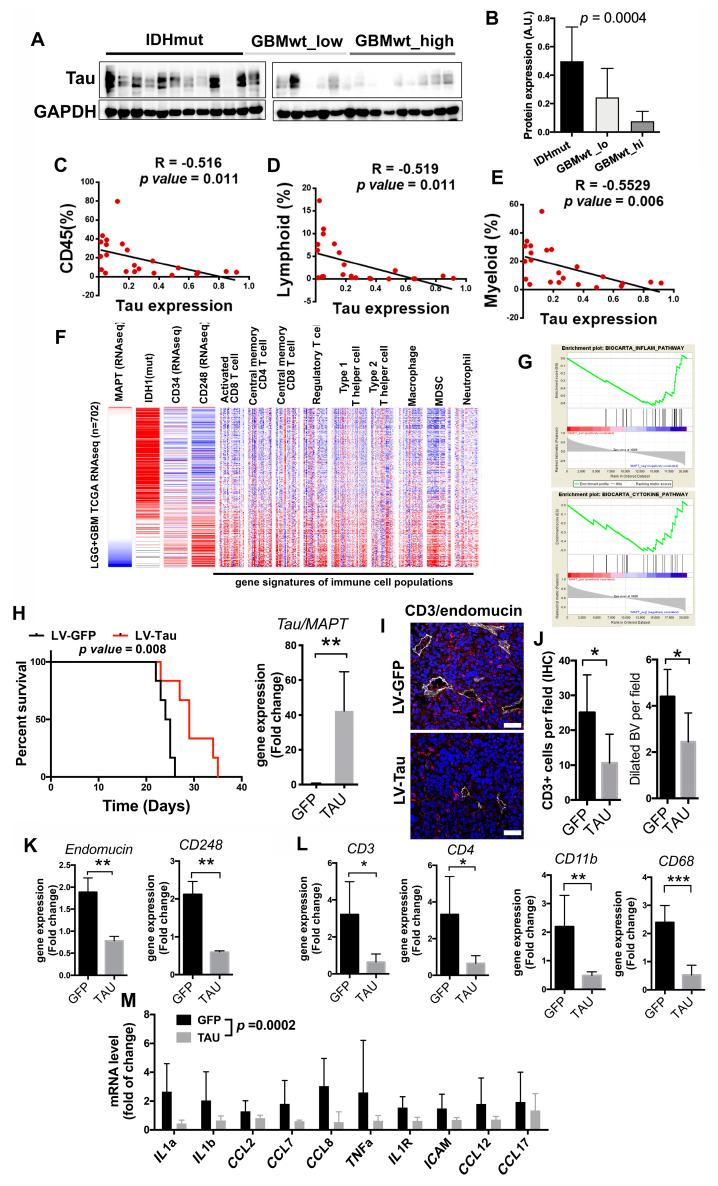
Tau expression correlates inversely with the immune content in gliomas. (**A**) Western blot (WB) analysis of Tau expression in extracts from IDHmut, GBMwt_lo and GBMwt_hi tumors. GAPDH level was used as a loading control. (**B**) Quantification of the WB in (**A**) (*n* = 26), the uncropped Western blots have been shown in Figure S8. (**C**–**E**) Correlation between the percentage of CD45+ (**C**), lymphoid (**D**) and myeloid (**E**) cells and Tau protein expression (circles represent each individual tumor) (*n* = 23). (**F**) Expression (RNAseq) of *IDH1*, *CD34*, *CD248* and 9 different immune population signatures in gliomas (the cancer genome atlas (TCGA) cohort). Samples were arranged according to their levels of expression of *MAPT/Tau* (*n* = 702). (**G**) Gene Set Enrichment Analysis (GSEA)-enrichment plot analysis using Tau gene expression values as template and the inflammatory and cytokine pathway gene set from the Biocarta pathways database. (**H**) Kaplan–Meier overall survival curves of C57Bl/6 mice that were orthotopically injected with GL261 cells that overexpressed Tau or GFP proteins (*n* = 5). Quantification of the amount of *Tau/MAPT* transcription in the tumors is shown on the right. (**I**) Representative pictures of CD3 (red) and endomucin (gray) staining in tumors from (**H**). (**J**) Quantification of the number of CD3^+^ cells and the number of dilated BVs per field in (**I**). (**K**–**L**) Expression of vascular (**K**) and immune (**L**) genes in the GL261 tumors (**H**) (*n* = 4). (**M**) Expression of cytokines and chemokynes in the GL261 tumors (**H**) (*n* = 4), the statistical analysis used for the global comparison was paired *T*-test. Scale bar: 10μm. * *p* < 0.05, ** *p* < 0.01, *** *p* < 0.001, ns: not significant.

We have recently described that Tau, a protein associated with neurodegenerative diseases, is also expressed in glioma cells, particularly in the less aggressive tumors, where it obstructs glioma progression by blocking the formation of novel and aberrant tumor vessels. These effects were associated with a limited capacity of the gliomas cells to contribute to the pool of pericytes in Tau-high tumors, which results in a reduced number of dilated BVs and a less efficient fueling of tumor growth [11]. We measured the amount of Tau in our cohort of gliomas and we observed that it accumulates in IDHmut gliomas (Figure 5A,B). This result was not surprising given that the *MAPT/Tau* gene is epigenetically induced by the presence of mutant IDH proteins [11]. However, we also found an enrichment of Tau in GBMwt_lo compared to GBMwt_hi tumors (Figure 5A,B). Moreover, we found an inverse correlation between the levels of Tau protein and the immune content of gliomas (Figure 5C–E). The in silico analysis of the TCGA database also revealed a strong inverse correlation between the amount of *MAPT/Tau* transcription and overall survival or the expression of vascular- (*CD34* and *CD248*) (Figure 5F and Figure S6A,B) and immune- (*CD3*, *CD4*, *CD11b* and *CD68*) (Figure 5F and Figure S6C–F) related genes. Notably, the transcript levels of *MAPT/Tau* and *CD248* were inversely and directly correlated, respectively, with several of the signatures associated with different immune cell populations (Figure 5F) and with the inflammatory- and cytokine-related pathways (Figure 5G). We also analyzed which genes were downregulated in Tau-high gliomas and we found that many of them were linked to the immune response (Figure S6G). Altogether, these results suggest that Tau could modulate the immune landscapes of gliomas.

To obtain further insight into the function of Tau in the glioma microenvironment, we overexpressed this protein in GL261 cells, a well-known mouse glioma model. Tau overexpression reduced tumor growth (Figure S6H) and increased survival (Figure 5H) of mice bearing orthotopic tumors, which is in agreement with the increased survival of patients with low *MAPT/Tau* expression (Figure S6I). The analysis of the tumors revealed a decrease in the amount of infiltrating CD3 lymphocytes, in parallel with a reduction in the number of dilated BVs in the Tau-overexpressing gliomas (Figure 5I–J). The transcriptomic analysis of the tumor tissues confirmed the inhibition of the expression of vascular- (Figure 5K) and immune- (Figure 5L) related genes in GL261-Tau tumors, compared to their control counterparts. Moreover, the expression of Tau reduced a signature of cytokines and chemokines (Figure 5M) already described as inducers of immune recruitment in brain tumors (Figure 5G) [18]. In agreement with that, we found a decrease in the amount of CD11b upon Tau overexpression, with no changes in the levels of IBA1 protein (Figure S6J). These findings support the idea that Tau modulates both the vascular features of gliomas and the entrance of immune cells.

We have previously shown that the Tau expression is induced by IDH mutations and repressed by wild-type IDH1 [11]. In agreement with these data, we found that total *IDH1* expression was upregulated in those GBMs with a higher immune content (Figure S7A). This result suggests an explanation for the downregulation of Tau expression in the GBMwt_hi subgroup, which could be responsible, at least in part, for the increase in the vascular abnormalities and with the immune-enriched TME observed in these gliomas. However, we cannot discard that epigenetic changes induced by a higher amount of wild-type IDH could be affecting the expression of other immunomodulatory molecules as well. One such gene could be *HLA-A*, whose expression can be modulated by epigenetic mechanisms [19]. As a matter of fact, we observed a decrease in the amount of *HLA-A* transcription in the G-CIMP gliomas (Figure S7B), a phenotype associated with the presence of IDH mutations. When we analyzed our cohort, we observed that *HLA-A* transcription was elevated in the GBMwt_hi subgroup, in comparison with the rest of gliomas (Figure S7C). Moreover, we found a strong correlation between the expression of *HLA-A* and *IDH1*wt in the TCGA dataset (Figure S7D), similar to the one observed between *MAPT*/Tau and *IDH1*. Taken together, our results suggest that the balance between mutant and wild-type IDH function in gliomas is controlling the expression of Tau, and probably other proteins, to shape the vascular and the immune niche of gliomas.

## 4. Discussion

A recent pan-cancer immunogenomic analysis has emphasized the unique microenvironment of glioma with an enrichment of the lymphocyte-depleted and the macrophage-enriched signatures in GBMs, whereas LGGs showed an immunologically quiet (“cold”) expression pattern [4]. Our detailed characterization of the immune content of different gliomas suggests that the presence of *IDH1/2* mutations, even more than the histological grading, is the best predictor of a reduced immune infiltration in gliomas. This observation is in agreement with the results obtained in mouse models [14] and with the retrospective analysis of human data [7,8,20,21,22]. The paucity of immune cells in IDHmut gliomas could participate in the reduced aggressiveness of IDHmut gliomas, as leukocytes facilitates tumor proliferation [23]. As a drawback, tumors bearing IDH mutations could have an inferior response to immunotherapy. For that reason, several clinical trials have been designed combining inhibitors of mutant IDH with ICIs and vaccinations [24], a strategy that has proven to be effective in preclinical models [21,22]. However, it remains a pending task to prove that anti-tumor leukocytes can enter IDHmut gliomas in an efficient way.

GBM, even if we exclude IDHmut tumors, is not a homogenous entity. Here, we have described different levels of immune extravasation in IDHwt GBMs, which has allowed us to classify these tumors based on the presence of high or low levels of leukocytes. Notably, the expression of PN or MES markers did not allow us to distinguish between these two immune GBM subgroups. These results differ from some computational [7] and IHC [9,10] studies, showing the enrichment of myeloid and lymphoid cells in the MES subtype. Another study, however, observed an accumulation of CL tumors among GBMs with a high immune infiltrate [8]. In any case, we have recently observed at least two subgroups of CL GBMs and a poor molecular definition of MES tumors based on molecular alterations [25], suggesting a blurred frontier between these two groups of aggressive gliomas.

The immune compartment of the GBMwt_high subgroup can account for half of the tumor mass, basically at the expense of recruited cells. Notably, no clear differences were found in the amount of microglia amid the different subgroups. Our results are in agreement with recently published data showing that the main immune signature in IDHmut gliomas corresponds to microglia, whereas in the wild-type tumors there is an accumulation of infiltrating myeloid and T cells [26,27]. Notably, we have found a high level of expression of CD206, a marker of pro-tumoral M2 macrophages, in myeloid cells in the GBMwt_high tumors, and a strong increase in PD-L1 expression between this subgroup and the rest of gliomas. This increment is mostly due to its presence on the surface of the immune cells, where myeloid cells are enriched. Although we cannot discard the presence of PD-L1 in lymphocytes [28], its expression has already been described in GBM infiltrating macrophages [29]. Based on our results, strategies to impair the high immunosuppressive environment of GBMwt_hi tumors are essential. In agreement with that, it has been recently reported that targeting of myeloid cells increases the response to anti-PD-1 in glioma mouse models [30]. By contrast, the low PD-L1 expression in the rest of gliomas could be hampering the result of antibodies targeting this molecule. However, the lower CD4/CD8 ratio and the scarcity of myeloid cells in the GBMwt_lo gliomas could make them more prone to respond to different immunotherapies not only based on the PD-1–PD-L1 axis. In any case, our results highlight the relevance of a patient selection, reasonably based on the vascular–immune profile, to improve the success of future immunotherapies.

Several groups have attributed the scarcity of T cells in IDHmut gliomas to the accumulation of the oncometabolite 2-hydroxyglutarate [14,21,22,24]. However, these mutations could be affecting other components of the TME, specially macrophages and MDSCs through different mechanisms, such as implementing a specific cytokine program as it has been discovered in IDH mutant gliomas [31]. Here, we propose that, secondary to IDH mutations and/or to a reduction in the amount of wild-type IDH enzymes, Tau accumulates in the tumors. By contrast, GBMwt_hi gliomas, which contain the higher expression of IDHwt, had a reduced amount of Tau. It is important to point out that the balance of wild-type and mutant IDH proteins controls the clinical outcome of gliomas, including their sensitivity to radiation and chemotherapy [32]. Notably, the wild-type isoform of this gene is upregulated in primary GBMs and promotes aggressive growth and therapy resistance [33]. Moreover, it has been shown that IDHwt expression reshapes the methylome and also affects gene expression [34], which could explain Tau downregulation. In agreement with our previous results [11], we have shown here that as the level of Tau decreases, the number of pericytes increases and the tumor vasculature appears distorted, with numerous enlarged vessels. Moreover, we have observed a striking positive correlation between the presence of the immune infiltrate and the appearance of these vascular abnormalities. Additionally, and in agreement with our hypothesis, the levels of Tau decreased in parallel to these changes in the immune profiles. Furthermore, the analysis of different orthotopic mouse and human glioma models has confirmed that overexpression of Tau reduces the amount of both leukocytes and myeloid cells in the tumors, in parallel to changes in the cytokine and inflammatory signatures as well as with the normalization of the vessels.

Regarding clinical implications, there is evidence of a possible correlation between the presence of innate immunity cells and the aggressive behavior of gliomas [6]. However, our survival analysis could not detect significant differences in the clinical behavior of the two IDHwt GBM subgroups, as other investigators have shown [20]. Although our study is conditioned by the limited number of cases to test survival differences, these results suggest that tumors with a low immune content do not represent a less aggressive entity that would progress into a GBMwt_hi tumor. This is in agreement with their similar appearance at MRI or in the macro-vascular analysis. Alternatively, changes in the cellular composition of the vessels and/or in the expression of adhesion molecules could determine the entrance of the immune cells in the different GBMs. In any case, our results underline the intricate connection between the two main components of the glioma niche. In relation to that, the main angiogenic factor, vascular endothelial growth factor (VEGF), can inhibit the function of T cells and increase the recruitment of regulatory Tregs and MDSCs [35]. Moreover, it has been shown that pericytes can support tumor growth via immunosuppression [36]. However, once in the tumors, the immune cells can also induce changes in the vascular compartment as they have a strong pro-angiogenic role [37,38]. Our data do not allow us to discriminate which came first: the chicken or the egg. In the presence of low levels of Tau, an increase in the number of glioma-derived pericytes and the subsequent changes in the blood–brain barrier (BBB), could directly ease the extravasation of hematopoietic cells. However, we cannot discard that the downregulation of Tau might inhibit directly the secretion of molecules that attract myeloid cells, and this could further contribute to the vascular phenotype. Moreover, other proteins epigenetically induced or repressed by the balance between mutant and wild-type IDH will probably participate in the formation of the different immune–vascular landscapes. In any case, our results suggest that combined strategies targeting these two components of the tumors´ stroma might lead to promising results, as it is already being tested in recurrent GBMs, using nivolumab and bevacizumab (NCT03452579, NCT03743662, NCT03890952). However, based on our results, an effort should be made to understand which group of patients could benefit most from these combinatorial treatments and also to design new treatment schemes.

## 5. Conclusions

Our results show that there is a reduced infiltration of immune cells in IDH mutant gliomas. By contrast, the immune profile of their wild-type counterparts is more heterogeneous, with some tumors highly enriched in immunosuppressive cells (with strong PD-L1 expression) and others with few lymphocytes and myeloid cells. The cellular and molecular characterization of gliomas revealed a direct correlation between the presence of vascular alterations and the arrival of leukocytes into the tumors. By contrast, both features inversely correlated with the levels of the microtubule-binding protein, Tau. We propose that the expression of Tau, which is governed by the *IDH* genetic status, regulates the vascular and the immune content of gliomas simultaneously. These results highlight the strong connection between the two compartments of the glioma microenvironment and suggest that synergistic approaches should be considered.

## Figures and Tables

**Table 1:** Characteristics of the study population. KPS: Karnofsky Performance Scale; ATRX: α-thalassemia/mental retardation syndrome X-linked; IDH: isocitrate dehydrogenase; MGMT: O6-methylguanine-DNA-methyltransferase; w/t: wild type.

*n*	28
Age (years)	
Median	52 years
Range	30–82 years
Gender	
Female	n = 12; 43%
Male	n = 16; 57%
Grade of resection	
Complete	n = 23; 82%
Partial	n = 5; 18%
KPS after surgery	
100	n = 10; 36%
90	n = 9; 32%
80	n = 4; 14%
70	n = 5; 18%
Histological Diagnosis	
Astrocitoma	n = 25; 89%
Oligodendroglioma	n = 3; 11%
Tumor grade	
II	n = 3; 11%
III	n = 6; 22%
IV	n = 19; 67%
IDH 1/2	
Mutated	n = 10; 36%
w/t	n = 18; 64%
ATRX	
Mutated	n = 8; 28%
w/t	n = 21; 72%
MGMT	
Methylated	n = 21; 72%
Unmethylated	n = 2; 7%
Unknown	n = 5; 21%
TERT promoter	
Mutated	n = 19; 62%
Wild Type	n = 10; 34%
Unknown	n = 1; 4%
1st line therapy	
Stupp (RT + temozolomide)	n = 21; 75%
Temozolomide	n = 1; 4%
RT + PCV	n = 2; 7%
None	n = 4; 14%

## Supplementary Materials

The following are available online at https://www.mdpi.com/2072-6694/12/11/3230/s1, Figure S1: Representative plots of the gating on FACS to identify leukocytes and myeloid cells in human glioma tissues. Figure S2: Sequencing and Overall survival in de glioma cohort, Figure S3: Microglia content is similar in IDHmut, GBMwt_lo, GBMwt_hi gliomas, Figure S4: Proneural or mesenchymal markers expression in IDHmut, GBMwt_lo, GBMwt_hi gliomas, Figure S5: Positive correlation between CD34^+^ and CD248^+^ or CD45^+^ cells content in gliomas, Figure S6: Inverse correlation between Tau expression and vascular alterations or immune cells content in gliomas, Figure S7: Epigenetic changes could explain differences between the groups, Figure S8: Uncropped Western blots, Table S1: Study patient´s characteristics, Table S2: List of antibodies used for flow cytometry (FACS), Western blot (WB) and immunohistochemistry (IHC) analysis, Table S3: List of primers used for qRT-PCR analysis.

## Abbreviations

ATRX	α-thalassemia/mental retardation syndrome X-linked
BBB	blood–brain barrier
BV	blood vessels
CGP	chemical and genetic perturbations
CL	classical
FFPE	formalin-fixed paraffin-embedded
CP	canonical pathways
GSEA	gene set enrichment analysis
GBM	glioblastoma
GBMwt_hi	a subgroup of IDH wild-type tumors with a high content of CD45+ cells (more than 15% of the cellular suspension)
GBMwt_lo	a subgroup of IDH wild-type tumors with a low content of CD45+ cells (less than 15% of the cellular suspension)
IBA1	ionized calcium binding adaptor molecule 1
ICI	immune checkpoint inhibitors
IDH1/2	isocitrate dehydrogenase 1/2
IHC	immunohistochemistry
LGG	lower-grade glioma
MDSC	Myeloid-derived suppressor cells
MES	mesenchymal
MGMT	O6-methylguanine-DNA-methyltransferase
mut	mutant
ns	not significant
PCR	polymerase chain reaction
PD-1	programmed cell death 1
PD-L1	programmed death ligand 1
PN	proneural
RT	room temperature
SEM	standard error of the mean
TCGA	the cancer genome atlas
TME	tumor microenvironment
Tregs	regulatory T cells
VEGF	vascular endothelial growth factor
wt	wild-type
