# Supplementary material for: Immune Profiling of Gliomas Reveals a Connection with IDH1/2 Mutations, Tau Function and the Vascular Phenotype
Source: Cancers (Basel). 2020 Nov 2;12(11):3230. doi: 10.3390/cancers12113230 (PMC7694073; doi:10.3390/cancers12113230)
Supplement: Supplementary file 1 [file cancers-12-03230-s001.pdf]

Supplementary Materials: Immune Profiling of Gliomas Reveals a Connection with IDH1/2 Mutations, Tau Function and the Vascular Phenotype.

Teresa Cejalvo, Ricardo Gargini, Berta Segura-Collar, Pablo Mata-Martínez, Beatriz Herranz, Diana Cantero, Yolanda Ruano, Daniel García-Pérez, Ángel Pérez-Núñez, Ana Ramos, Aurelio Hernández-Laín, María Cruz Martín-Soberón, Pilar Sánchez-Gómez and Juan M. Sepúlveda-Sánchez

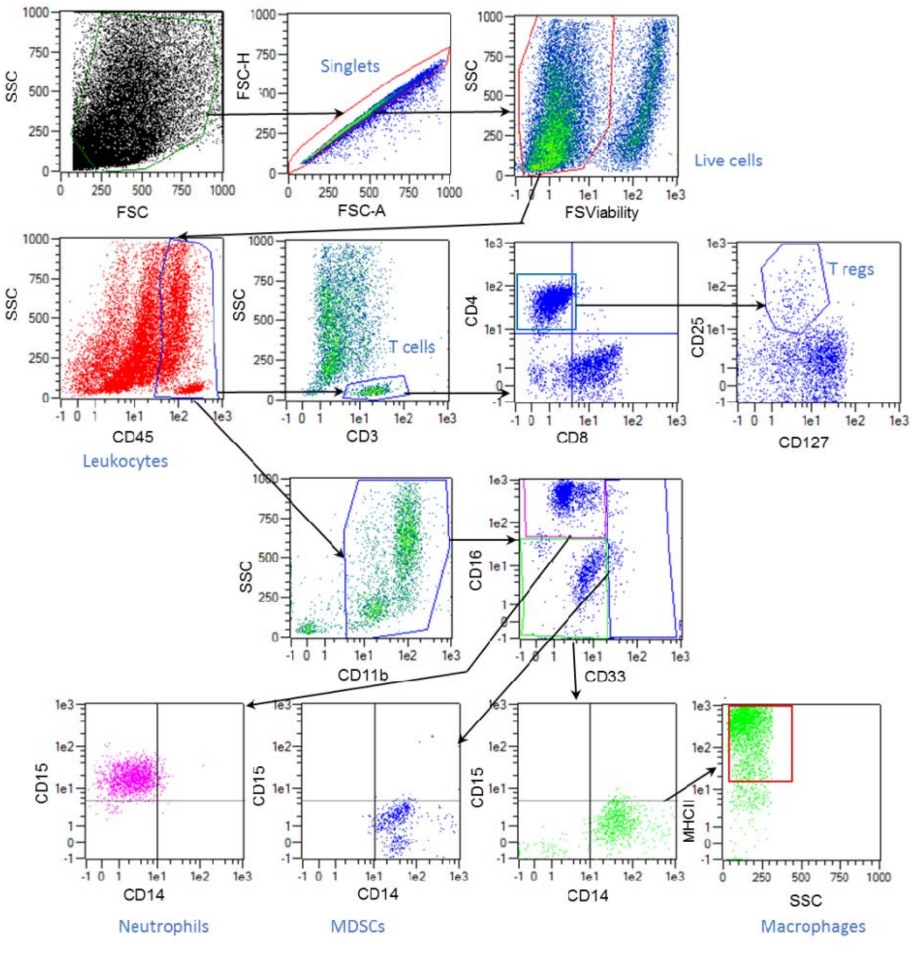

Figure S1. Representative plots of the gating on FACS to identify leukocytes and myeloid cells in human glioma tissues. A Fixable Staining for Viable cells detection has been used for excluding dead cells.

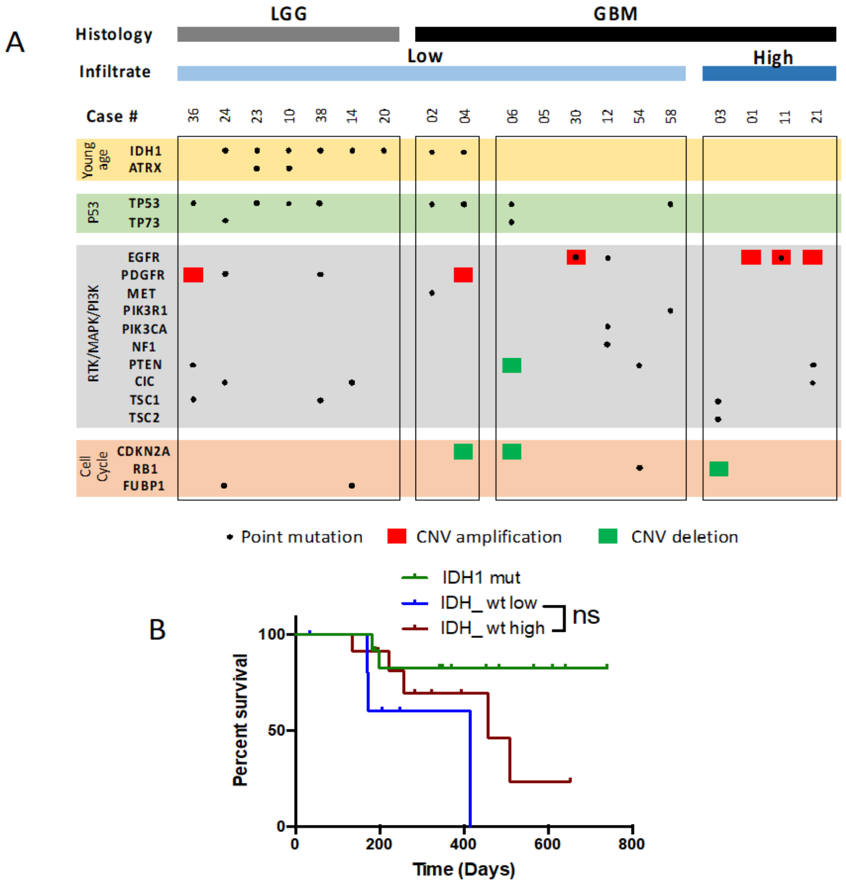

Figure S2. Sequencing and Overall survival in de glioma cohort (**A**) Distribution of point mutations and copy number variations in 20 genes frequently altered in gliomas. (**B**) Kaplan-Meier survival curves of the three different groups associated with the immune phenotype, IDHmut, GBMwt_lo, GBMwt_hi gliomas.

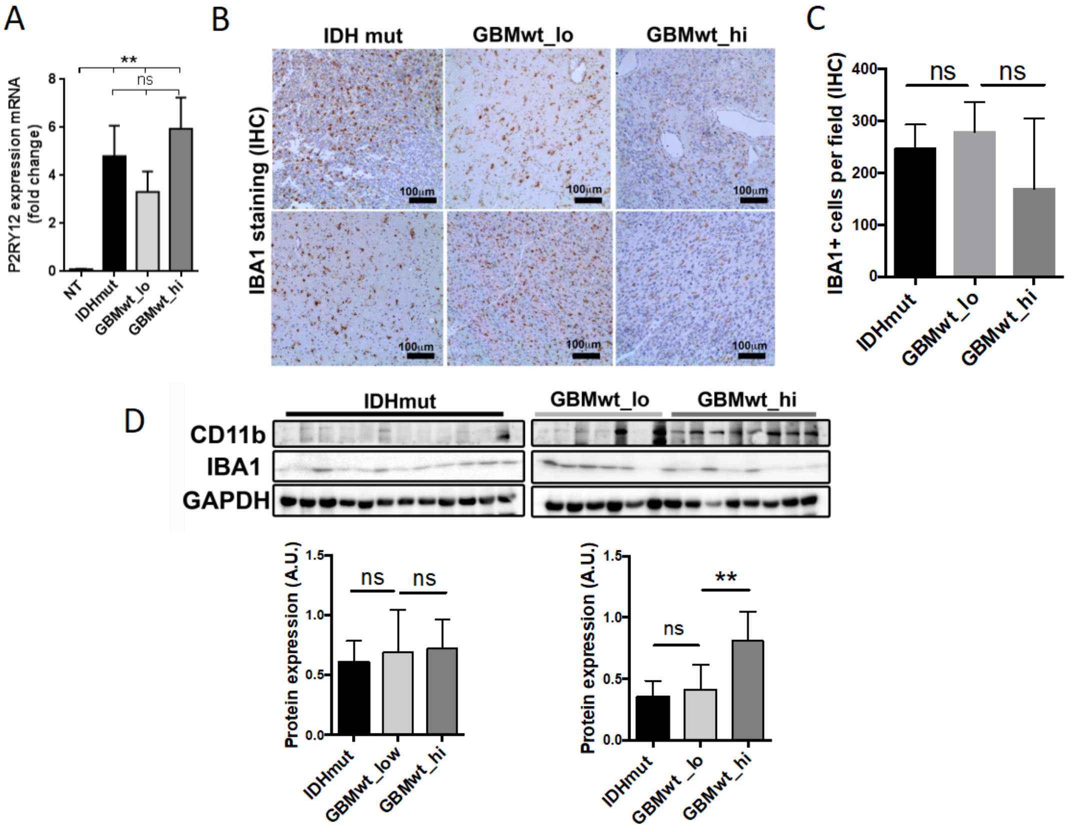

Figure S3. Microglia content is similar in IDHmut, GBMwt_lo, GBMwt_hi gliomas. (**A**) Fold change values of qRT-PCR analysis of P2RY12 expression in tumor tissue from IDHmut, GBMwt_lo, GBMwt_hi (n = 6) NT (normal brain tissue). (**B**) Representative pictures obtained from IHQ for IBA1 in the three groups of gliomas. (**C**) Quantification of IBA1 positive cells per field using conventional microscope at 20× from IHQ showed in B (n = 6). (**D**) WB analysis of CD11b and IBA1 expression in tumor tissue extracts from IDHmut (black), GBMwt_lo (light grey), GBMwt_hi (dark grey) tumors. GAPDH level as a loading control. (**E**) Quantification of levels of CD11b and IBA1 expression from WB showed in D. ** $p < 0.01$, ns not significant. Scale bars: 100 µm.

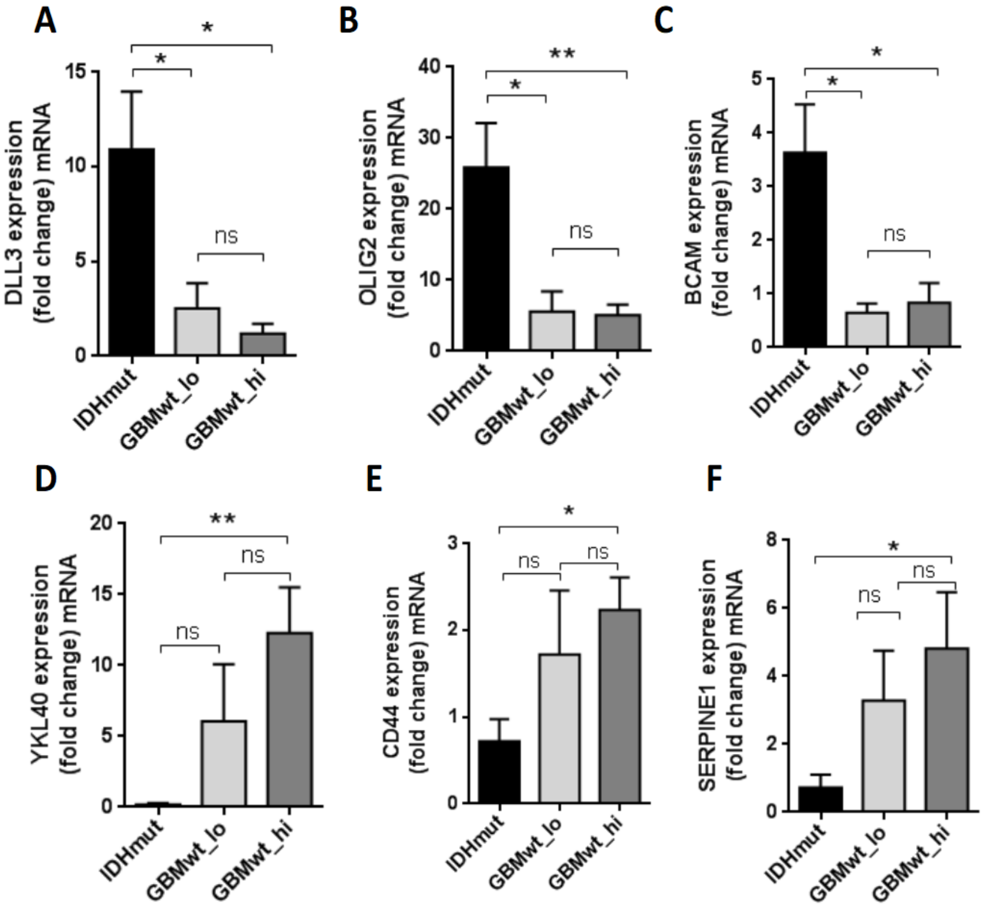

Figure S4. Proneural or mesenchymal markers expression in IDHmut, GBMwt_lo, GBMwt_hi gliomas. Fold change values of qRT-PCR analysis of expression of (**A**–**C**) typical proneural markers as *DLL3*, *OLIG2* and *BCAM*. (**D**–**F**) typical mesenchymal markers as *YKL40*, *CD44* and *SERPINE1*. *HPRT* was used for normalization ($n = 26$). * $p < 0.05$, ** $p < 0.01$, ns not significant.

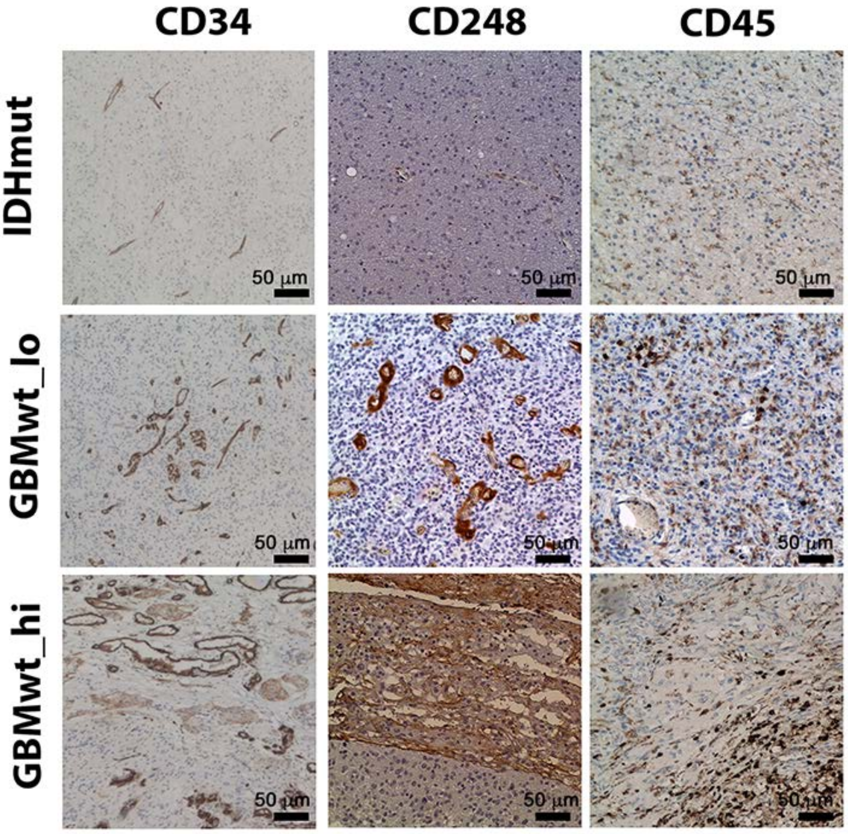

Figure S5. Positive correlation between CD34⁺ and CD248⁺ or CD45⁺ cells content in gliomas. Representative pictures from IHQs of CD45 (leukocyte marker), CD34 (endothelial marker) or CD248 (pericyte marker) in each group of gliomas. Scale bars: 50 µm.

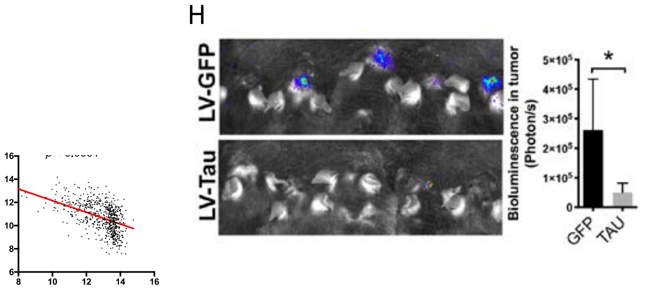

Figure S6. Inverse correlation between Tau expression and vascular alterations or immune cells content in gliomas. Correlation of the mRNA expression by RNAseq of *Tau (MAPT)* with that of (**A**) CD34 (**B**) CD248 (**C**) CD3E (**D**) CD4 (**E**) CD11b and (**F**) CD68 in gliomas using the TCGA cohort (n = 661). (**G**) Top enriched Gene Ontology (GO) biological process for the cluster of 500 genes which expression is negatively correlated with Tau (MAPT) expression in gliomas. LGG+GBM merge cohort

and the DAVID gene ontology program are used. (**H**) Representative image and quantification of tumor growth using IVIS ILUMINA through luciferase-luciferin system. (**I**) Kaplan-Meier overall survival curves of patients from the TCGA cohort glioma IDH wt ($n = 191$) and patients in each cohort were stratified into 2 groups based on high and low *Tau/MAPT* expression values, log-rank (Mantel-Cox) test. (**J**) WB analysis of CD11b and IBA1 expression in tumor tissue extracts from GFP and Tau-GL261. GAPDH level as a loading control and quantification of levels of CD11b and IBA1 expression. * $p < 0.05$, ** $p < 0.01$, ns not significant.

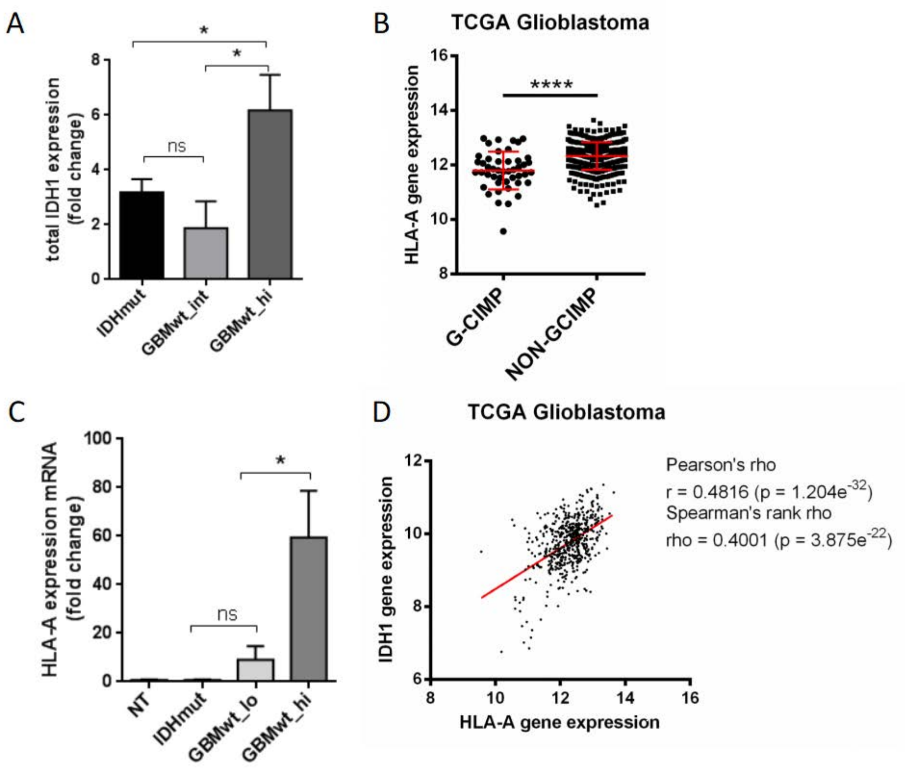

Figure S7. Epigenetic changes could explain differences between the groups. **(A**) Fold change values of qRT-PCR analysis of *IDH1* expression ($n = 6$). (**B**) Analysis of *HLA-A* mRNA expression by RNAseq in gliomas (TCGA cohort) grouped according to their G-CIMP status (G-CIMP (big circles represent individual tumors) $n = 46$ and NON-GCIMP (small circles represent individual tumors) $n = 475$). (**C**) Fold change values of qRT-PCR analysis of *HLA-A* expression ($n = 6$). Normal Tissue (NT). (**D**) Correlation of the expression mRNA of *IDH1* and *HLA-A* using TCGA glioma cohort ($n = 539$). Paired T-test was performed. * $p < 0.05$, **** $p < 0.0001$, ns not significant.

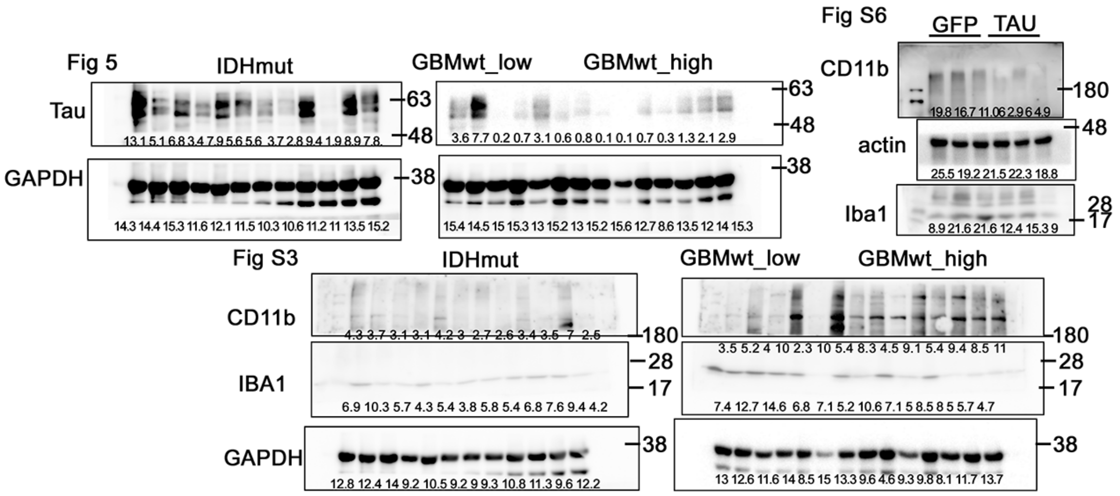

Figure S8. Uncropped western blots.

Table S1. Study patient´s characteristics.

Patient	Age	Gender	Grade of Resection	Diagnosis	Tumor Grade	*IDH*	*ATRX*	Immune Profile	*MGMT*	*TERT*	1st Line Treatment	Time to PD	Time of F/U	Status After F/U
G001	82	female	complete	astrocitoma	IV	WT	WT	GBMwt_hi	methylated	C228T	Stupp	4.5	7.6	death
G002	36	female	complete	astrocitoma	IV	Mut	Mut	IDHmut or LGG	methylated	wt	Stupp	No PD	25	alive
G003	68	male	complete	astrocitoma	IV	WT	Mut	GBMwt_hi	methylated	wt	Stupp	8.2	8.9	death
G004	42	male	complete	astrocitoma	IV	Mut	Mut.	IDHmut or LGG	methylated	wt	Stupp	4.3	5.5	death
G005	70	male	complete	astrocitoma	IV	WT	WT	GBMwt_lo	methylated	C228T	none	4.6	4.6	death
G006	57	male	complete	astrocitoma	IV	WT	WT	GBMwt_lo	methylated	C228T	none	1	1	death
G007	34	female	complete	astrocitoma	IV	WT	WT	GBMwt_hi	methylated	wt	Stupp	16.7	23	death
G010	30	female	complete	astrocitoma	III	Mut	Mut	IDHmut or LGG	methylated	wt	Stupp	No PD	22.4	alive
G011	52	male	complete	astrocitoma	IV	WT	WT	GBMwt_hi	unmethylated	C228T	Stupp	17.3	21.6	alive
G012	53	male	partial	astrocitoma	IV	WT	WT	GBMwt_lo	methylated	C228T	Stupp	6.6	13.6	death
G014	30	female	partial	oligodendroglioma	II	Mut	WT	IDHmut or LGG	methylated	C228T	RT+PCV	No PD	20	alive
G020	46	male	complete	oligodendroglioma	II	Mut	WT	IDHmut or LGG	methylated	C228T	RT+PCV	No PD	18.9	alive
G021	55	female	complete	astrocitoma	IV	WT	WT	GBMwt_hi	methylated	wt	Stupp	6.6	14.7	death
G023	45	female	complete	astrocitoma	III	Mut	Mut	IDHmut or LGG	methylated	wt	Stupp	No PD	14.9	alive
G024	35	female	complete	oligodendroglioma	II	Mut	WT	IDHmut or LGG	methylated	C228T	none	No PD	16.8	alive
G030	63	male	complete	astrocitoma	IV	WT	WT	GBMwt_lo	methylated	C228T	Stupp	5.5	5.5	death
G035	65	female	complete	astrocitoma	IV	WT	WT	GBMwt_hi	no data	C228T	Stupp	4	14.2	alive
G036	76	female	partial	astrocitoma	III	WT	WT	IDHmut or LGG	methylated	C228T	temozolomide	4.1	4.2	death
G038	39	male	complete	astrocitoma	III	Mut	Mut	IDHmut or LGG	no data	wt	Stupp	No PD	13.3	alive
G039	47	male	complete	astrocitoma	IV	Mut	Mut.	IDHmut or LGG	methylated	wt	Stupp	No PD	13	alive
G043	38	male	partial	astrocitoma	III	Mut	Mut.	IDHmut or LGG	methylated	C228T	Stupp	No PD	12	alive
G045	65	male	complete	astrocitoma	IV	WT	WT	GBMwt_hi	no data	no data	Stupp	No PD	11	alive
G048	69	female	complete	astrocitoma	IV	WT	WT	GBMwt_hi	no data	C228T	Stupp	9.3	10.6	alive
G054	42	male	complete	astrocitoma	IV	WT	WT	GBMwt_lo	no data	wt	Stupp	No PD	9.7	alive
G055	71	female	partial	astrocitoma	IV	WT	WT	GBMwt_hi	methylated	C228T	none	4.4	4.4	death
G058	70	male	complete	astrocitoma	IV	WT	WT	GBMwt_lo	methylated	C250T	Stupp	No PD	7.6	alive
G059	50	male	complete	astrocitoma	III	WT	WT	IDHmut or LGG	unmethylated	C228T	Stupp	No PD	7.2	alive

G060	50	male	complete	astrocitoma	IV	WT	WT	GBMwt_hi	methylated	C228T	Stupp	No PD	7.1	alive

Table S2. List of antibodies used for flow cytometry (FACS), Western Blot (WB) and immunohistochemistry (IHC) analysis.

Purpose	Antigen	Conjugated	Catalog Number	Supplier	Clone
FACS	cd25	421	302629	Biolegend	BC96
	cd3	vg	563109	BD	UCHT1
	cd45	488	130-080-202	Miltenyi	REA747
	cd127	pe	561028	BD	HIL-7R-M21
	cd8	pecy5	565310	BD	SK1
	cd4	647	300520	Biolegend	RPA-T4
	pd1	421	562516	BD	EH12.1
	cd16	421	302037	Biolegend	3G8
	cd14	pe	130-110-577	Miltenyi	REA599
	cd15	vg	301910	Biolegend	HI98
	cd33	pecy5	366615	Biolegend	P67.6
	cd11b	647	130-098-087	Miltenyi	M1/70
	pdl1	vg	329713	Biolegend	29E.2A3
	cd206	pecy5	321121	Biolegend	15-2
	mhcii	421	562805	BD	G46-6
WB	CD11b	Purified	SAB1305652	Sigma	40TST
	IBA1	Purified	ab16588	Abcam	EPR 16588
	GAPDH	Purified	sc-47724	Santa cruz	0411
	Tau	Purified	577801	CALBIOCHEM	Tau-5
IHC	CD34	Purified	NCL-L-END	Leica	QBEND/10
	CD248	Purified	564994	BD	B1/35
	CD45	Purified	M0701	Dako	2B11 + PD7/26
	CD3	Purified	A0452	Dako	Polyclonal Rabbit
	endomucin	Purified	sc-65495	Santa cruz	(V.7C7)

Table S3. List of primers used for qRT-PCR analysis.

Genes	Forward (5´-3´)	Reverse (3´-5´)
hBCA	CTGTCTGGAGAGAACTGCGT	TCTTGCAGGTGTAGGACAGG
hCD248	AGACCACCACTCATTTGCCTGGAA	AGTTGGGATAATGGGAAGCGTGGT
hCD34	CCCTCAGTGTCTACTGCTGGTCT	GGAATAGCTCTGGTGGCTTGCA
hCD44	AGAAGGTGTGGGCAGAAGAA	AAATGCACCATTTCCTGAGA
hDLL3	AAACCTATGGGCTTGAGGAG	CGCTGAGTACAATCAGTGGAA
hEMCN	GCAAGCACTTCAGCAACCAGCC	GGATCTGCCTTCCAGCACATTC
hIBA1	CCCTCCAAACTGGAAGGCTTCA	CTTTAGCTCAGGTGAGTCTTGG
hIDH1	CTATGATGGTGACGTTGCAGTCG	CCTCTGCTTCTACTGTCTTGCC
hOLIG2	CGGCTTTCCTCTATTTTGGTT	GTTACACGGCAGACGCTACA
hP2RY12	TGCCAAACTGGGACCAGGACCA	TGGTGGCTTCTGGTAGCGATC
hSERPINE1	CATAGTGGAAGTGATAGAT	ACTCTGTTAATTCGTCTT
hYKL40	ACACCTGGGAGTGGAATGAT	AGTTCCATCCTCCGACAGAC
mCCL12	GCTACAGGAGAATCACAAGCAGC	ACGTCTTATCCAAGTGGTTTATGG
mCCL17	TTGTGTTCGCCTGTAGTGCATA	CAGGAAGTTGGTGAGCTGGTATA
mCCL2	AGGTCCCCTGTCATGCTTCTG	TCTCCAGCCTACTCATTGGG
mCCL7	CAGAAGGATCACCAGTAGTCGG	ATAGCCTCCTCGACCCACTTCT
mCCL8	GGGTGCTGAAAAGCTACGAGAG	GGATCTCCATGTACTCACTGACC
mCD248	TTGATGGCACCTGGACAGAGGA	TCCAGGTGCAATCTCTGAGGCT
mCD3	TCTCATTGCGGGACAGGATGGA	CCTTGGAGATGGCTGTACTGGT
mEMCN	GCACACACCATGTCACTGCTTC	CAGCGCGATAACCACAGGCAAA
mICA	AAACCAGACCCTGGAACTGCAC	GCCTGGCATTTCAGAGTCTGCT
mIL-1R	CTGTTGGTGAGGAATGTGGCTG	GGCTCAGGATAACAGGTCTGTC
mIL-1α	ACGGCTGAGTTTCAGTGAGACC	CACTCTGGTAGGTGTAAGGTGC
mIL-1β	TGACGGACCCCAAAAGATGA	TCTCCACAGCCACAATGAGT
mTNFα	GCCTCTTCTCATTCCTGCTTG	CTGATGAGAGGGAGGCCATT

Publisher's Note: MDPI stays neutral with regard to jurisdictional claims in published maps and institutional affiliations.

© 2020 by the authors. Licensee MDPI, Basel, Switzerland. This article is an open access article distributed under the terms and conditions of the Creative Commons Attribution (CC BY) license (http://creativecommons.org/licenses/by/4.0/).
